# Osteoclastogenesis in Local Alveolar Bone in Early Decortication-Facilitated Orthodontic Tooth Movement

**DOI:** 10.1371/journal.pone.0153937

**Published:** 2016-04-20

**Authors:** Ya-Wen Chen, Hai-Cheng Wang, Long-Hua Gao, Chang Liu, Yu-Xi Jiang, Hong Qu, Cui-Ying Li, Jiu-Hui Jiang

**Affiliations:** 1 Department of Orthodontics, Peking University School and Hospital of Stomatology, 22 South Zhongguancun Avenue, Haidian District, Beijing 100081, China; 2 Department of Stomatology of the First Hospital of Jiaxing, 1882 Zhonghuan South Road, Nanhu District, Jiaxing 314001, China; 3 Department of Pathology, School & Hospital of Stomatology, Tongji University, Shanghai Engineering Research Center of Tooth Restoration and Regeneration, Shanghai 200072, China; 4 Department of Orthodontics, College of Stomatology, Dalian Medical University, 9 Lushun South Road West, Lushun Port District, Dalian 116044, China; 5 Department of Orthodontics, Shandong University School of Stomatology, 44 Wenhua West Road, Lixia District, Jinan 250012, China; 6 Department of Stomatology, Binzhou Medical University, 346 Guanhai Road, Laishan District, Yantai 264003, China; 7 Central Laboratory, Peking University School and Hospital of Stomatology, 22 South Zhongguancun Avenue, Haidian District, Beijing 100081, China; Nanjing Medical University, CHINA

## Abstract

**Objective:**

In the current study, we aimed to investigate the effects of alveolar decortication on local bone remodeling, and to explore the possible mechanism by which decortication facilitates tooth movement.

**Materials and Methods:**

Forty rabbits were included in the experiment. The left mandible was subjected to decortication-facilitated orthodontics, and the right mandible underwent traditional orthodontics as a control. The animals were sacrificed on the days 1, 3, 5, 7 and 14, after undergoing orthodontic procedures. Tooth movement was measured by Micro-CT, and the local periodontal tissues were investigated using H&E, Masson's trichrome and tartrate-resistant acid phosphatase (TRAP) staining. The mRNA levels of genes related to bone remodeling in the alveolar bone were analyzed using real-time PCR.

**Result:**

On days 3, 5, 7 and 14, tooth movement was statistically accelerated by decortication (P < 0.05) and was accompanied by increased hyperemia. Despite the lack of new bone formation in both groups, more osteoclasts were noted in the decorticated group, with two peak counts (P < 0.05). The first peak count was consistent with the maximum values of ctsk and TRAP expression, and the second peak counts accompanied the maximum nfatc1 and jdp2 expression. The increased fra2 expression and the ratio of rankl/opg also accompanied the second peak counts.

**Conclusions:**

Following alveolar decortication, osteoclastogenesis was initially induced to a greater degree than the new bone formation which was thought to have caused a regional acceleratory phenomenon (RAP). The amount of steoclastogenesis in the decorticated alveolar bone was found to have two peaks, perhaps due to attenuated local resistance. The first peak count in osteoclasts may have been due to previously existing osteoclast precursors, whereas the second may represent the differentiation of peripheral blood mononuclear cells which came from circulation as the result of hyperemia.

## Introduction

Alveolar decortication-facilitated orthodontics is a category of new introduced techniques which combines alveolar decortications with orthodontic treatment [1, 2]. It is characterized by the procedure that only cortical bone is cut, perforated or mechanically altered while the spongy bone remains intact [3].

Alveolar decortication has been widely used in orthodontics. It was first used to facilitate the correction of maxillary protrusion in 1931 and, since that time, to accelerate tooth movement [2, 4]. Numerous case reports of corticotomy-accelerated orthodontic tooth movement have been published, with excellent outcomes. Recently, the mechanism underlying the acceleration has gained the interest of many scholars [5–7]. The accelerated tooth movement has been attributed to the regional acceleratory phenomenon (RAP), in which the surrounding bone density decreases early on after injury, which may decreased local resistance of movement [2, 7, 8]. Micro-CT (microcomputed tomography) scans of various animal models resemble post-fracture images with increased bone remodeling [9–11], and demonstrate that the local alveolar bone undergoes demineralization and remineralization successively after decortication [5, 12]. Therefore, alveolar decortication-facilitated orthodontics are known as “accelerated osteogenic orthodontics (AOO)” [10].

In traditional orthodontics, both bone absorption by compression and formation by tension contribute to the tooth movement [6, 13]. However, how decortication initiates bone remodeling early in AOO treatment requires further exploration. Decortication alters the expression of genes related to bone metabolism (such as rank, rankl, m-csf, TRAP, opn, and bsp) suggesting that either osteoclastogenesis or osteogenesis is involved in decorticated alveolar bone [6]. According to the rat model, in which tooth movement can be accelerated [5], decreased calcified spongiosa is accompanied by increased osteoclasts [14], which suggests the important role of bone resorption in decortication-facilitated orthodontics [7]. However, it is unknown whether osteogenesis or osteoclastogenesis is the primary contributor to the RAP and tooth movement acceleration; furthermore, the pattern of the osteogenesis or osteoclastogenesis remains unclear.

We know that the first step of tooth movement is bone resorption on the pressure side. In order to find out what happens in the beginning stages after decortication and eliminate interference factors, we focused primarily on osteoclasts in this study. We hypothesized that decortication accelerates osteoclastogenesis in rabbit local alveolar bone early in orthodontic tooth movement, and that this is associated with tooth movement [14].

## Materials and Methods

### Animal study

Decortication-facilitated animal models were established in forty female New Zealand white rabbits, 5 months old and weighing between 3.4 and 5 kg, all consistent with our previous study [1]. Rabbits were divided into five groups for different time points: the days 1, 3, 5, 7 and 14, post-decortication. At each time point, eight rabbits were sacrificed with an excess of pentobarbital sodium. Each of the animals were placed in a 60 x 40 x 40 cm cage without bedding materials, and then were acclimatized for 1 week before the experiments and then maintained at a controlled temperature (22 ± 2°C), with 12hr light/dark periods and free access to water and a commercial diet. Surgeries were performed under intravenous anesthesia (3% pentobarbital sodium, 1 ml/kg). This study was approved by the animal care committee guidelines of the Peking University biomedical ethics committee for laboratory animal welfare ethics (Beijing, China; permit number: LA2012-53).

### Surgical and orthodontic procedures

**Fig 1:**
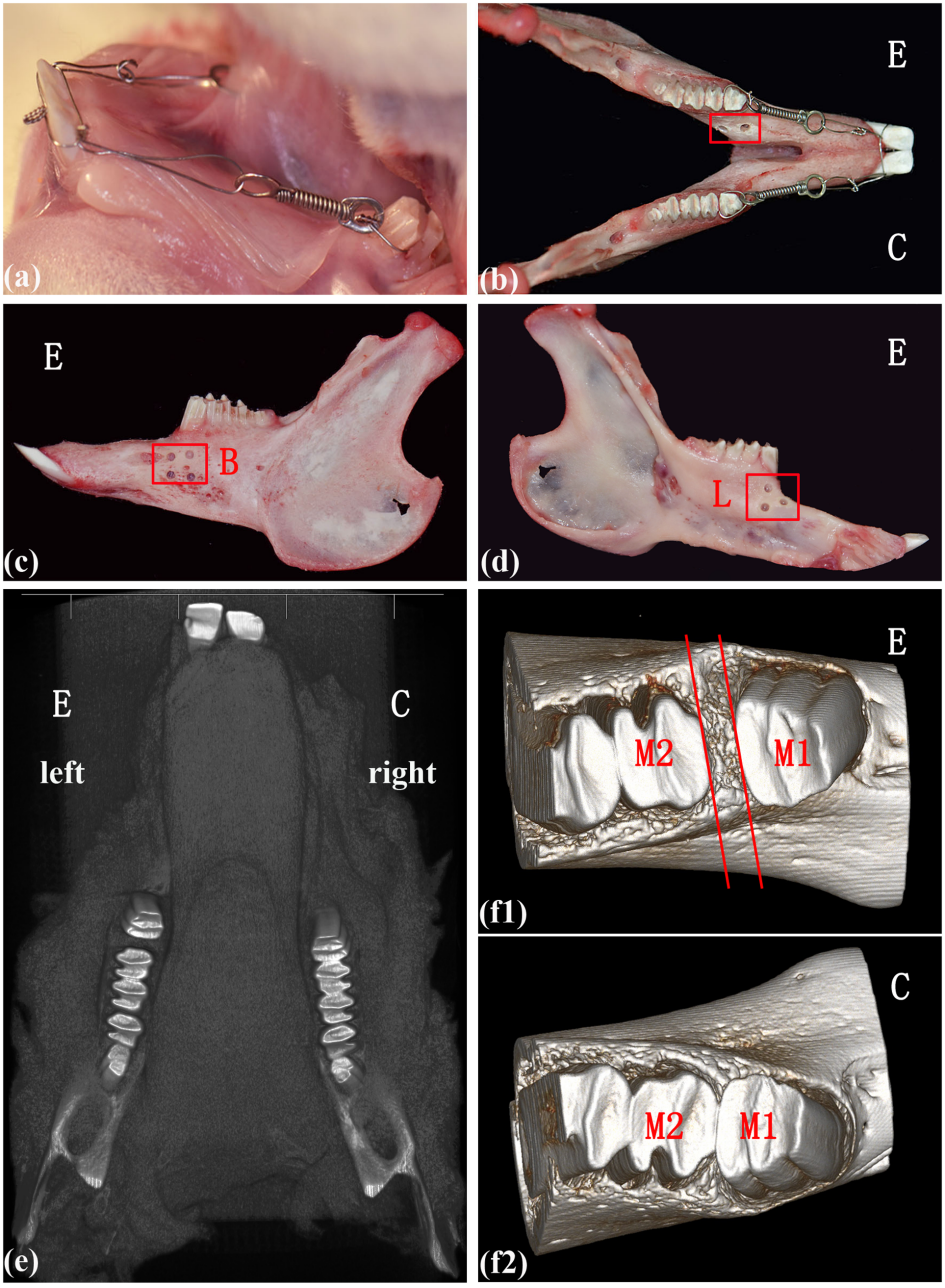
Alveolar decortication facilitates tooth movement. **(a-d)** The surgical and orthodontic procedures in the animal model, the red outline labelled the region of decortication; E-experimental group, C-control group, B-buccal side, L-lingual side. **(e-f2)** An illustration of the micro-CT used to measure the tooth movement distance; M1-the first molar, M2-the second molar.

Following the protocol of previous studies [6, 12, 15], the left mandibles were used as the experimental group, whereas the right side served as the control group (no surgical procedure). Before decortication, the rabbits were anesthetized by injecting sodium pentobarbital (30 mg/kg) into the lateral ear vein. After disinfection, a longitudinal incision (5 mm) was made over the alveolar ridge crest region, and mucosal flaps were folded back to expose the alveolar bone surface. Approximately 3 or 4 perforations (1-mm diameter, 1-mm depth) were then created on the buccal and lingual sides of cortical bone surface, made by a low-speed hand piece with copious water irrigation. After debridement, simple interrupted sutures were used to close the area. Immediately following, forces were applied to the mandibular first molars using superelastic NiTi (100 g; Sentalloy, Islandia, NY, USA) closed coil springs with eyelets (GAC International, Bohemia, NY, USA) between the mandibular first molars and incisors (Fig 1). Each rabbit was cared for after the surgery, and the iodine cotton ball was replaced daily until the wound had healed. No rabbits died from postoperative infections.

### Microcomputed tomography-assisted assessment of tooth movement

When the animals were sacrificed, at the appropriate time point, the mandibles were immediately isolated and imaged at a resolution of 50 microns with high-resolution Micro-CT, the Inveon MM CT (SIEMENS, Munich, Germany), as in previous studies [5, 6]. Samples were filtered to remove extraneous voxels with a Gaussian smoothing algorithm, and individually adjusted with a standard threshold algorithm to segment bone and nonbone voxels. Adjusted MicroCT images were used to measure the distance from the distal edge of the first molar to the mesial edge of the second molar (Fig 1). The measurements were made to the nearest 0.001 mm and were repeated at least three times.

### Histological investigation

Three mandibular segments (1 cm^3^ each), each containing the first molar and surrounding alveolar bone, were dissected for each time point. The samples were fixed in 10% buffered formalin at 4°C for 48 hours, rinsed with sterile saline, decalcified in 10% ethylenediamine tetra-acetic acid (EDTA-2Na, pH 7.4) for 2 months with daily replacement and embedded in paraffin. Five-micron thick sections were cut in the mesial-distal direction of the first molar. The sections were stained with hematoxylin and eosin (H&E). One section from each time point was stained with H&E, another section was stained with Masson’s trichrome stain to visualize the newly formed bone [16] and a third section was stained with tartrate-resistant acid phosphatase (TRAP; Sigma—Aldrich, St. Louis, MO, USA). TRAP-positive multi-nucleated cells (TRAP+MNCs) were considered osteoclasts [17, 18].

### RNA isolation and real-time PCR analysis

The expression of genes related to osteoclastogenesis markers were investigated. Five mesial alveolar bones from the first molar at each time point from each group were separated, and the total RNA was isolated with TRIZOL Reagent (Invitrogen, Grand Island, NY, USA). A total of 2 μg of total RNA was reverse-transcribed into cDNA using the Superscript First-Strand Synthesis System (Invitrogen) following the manufacturer’s protocol. Reactions were conducted in a 20-μl reaction mixture using the ABI 7500 real-time PCR system (ABI). The expression of genes related to bone metabolism was normalized to GADPH expression, expressed as 2^-(ΔCt)^. The primers for the osteoclastogenic markers in various stages (trap, ctsk, nfatc1, jdp2 and fra2), as well as regulators (rankl/opg) are listed in Table 1.

**Table 1:** The sequences of primers for real-time PCR.

Gene	Forward primer (5’-3’)	Reverse primers (5’-3’)
*trap*	gctacctccgcttccacta	gcagcctggtcttgaagag
*ctsk*	tggttcctgttgggctttca	cgggtaagcgtcttcagagtcaat
*nfatc1*	ctttatcagctgcacatcactcaga	cgctgggaacactcgatagg
*jdp2*	cgctgacatccgcaacatt	ggcctcttgcccagtttca
*fra2*	tcgccgggagctgaca	gcagctcagcaatctctttctg
*rankl*	agagcgaagacacagaagca	ccatcaatgctgccaacatc
*opg*	acggcggcatagttcacaag	cttcgcagcttgatggagag
*GAPDH*	tctcctgcgacttcaacagtg	ctcttactccttggaggccat

### Statistical analysis

All assays were repeated at least three times for each sample. Statistical analysis was performed using SPSS 18.0 (IBM, Armonk, NY, USA). T-tests were performed to evaluate the differences in data between the experimental and control groups. P < 0.05 was considered statistically significant.

## Results

### Alveolar decortication facilitated tooth movement

**Fig 2:**
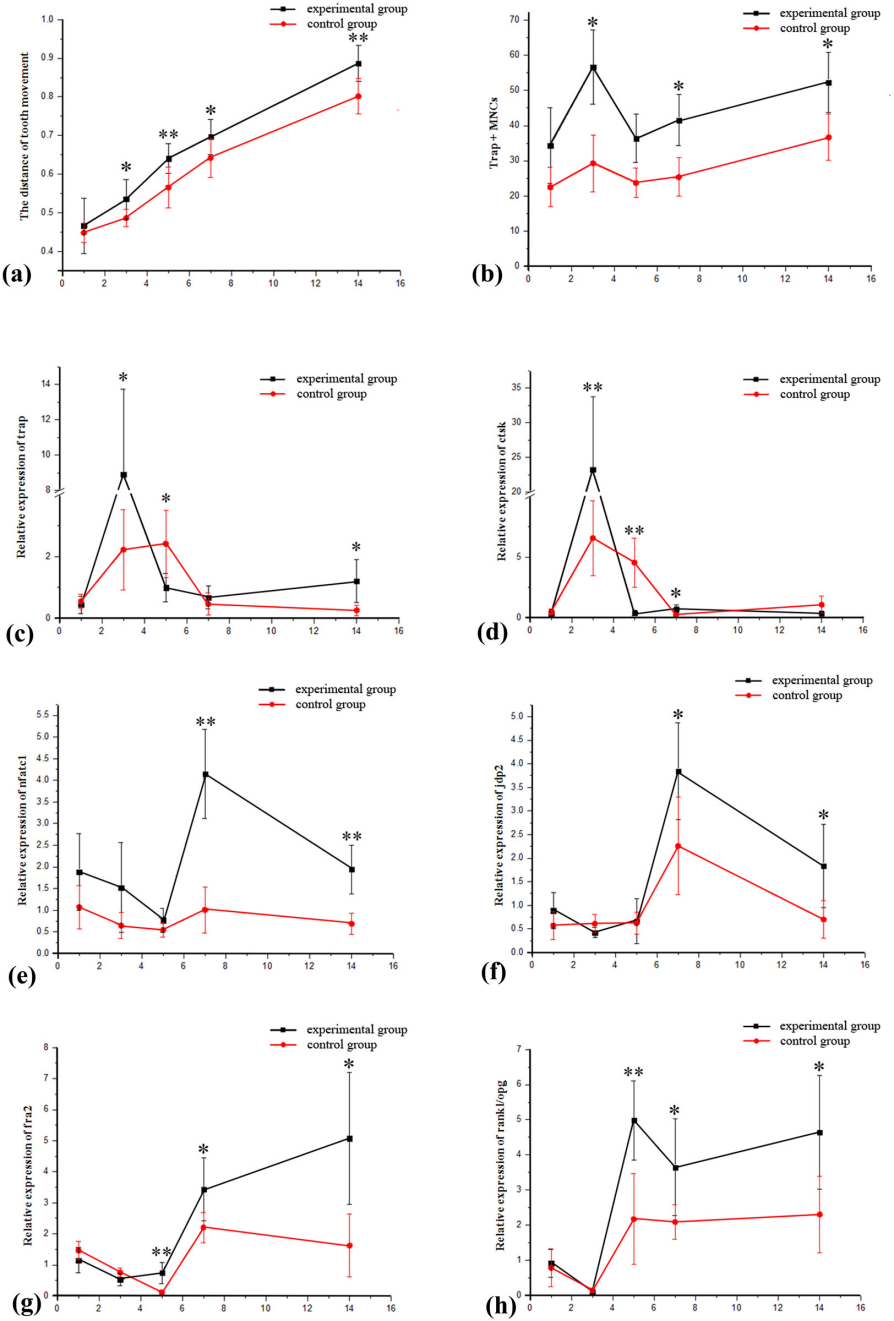
The changes and comparison between groups for tooth movement, osteoclasts and expression of genes at different time points. **(a)** The distances of tooth movement. **(b)** The osteoclast counts. **(c-h)** The mRNA levels of genes related to bone remodeling: ctsk, trap, nfatc1, jdp2 and fra2, as well as the ratio of rankl/opg. (*p < 0.05, **p < 0.01).

The tooth movement distance was measured with Micro-CT (Fig 1). The distances for the experimental groups (decorticated) were significantly greater than those for the control groups (P < 0.05) on days 3, 5, 7 and 14, after the orthodontic procedure (Fig 2; Table 2). This finding is consistent with previous studies and supports the establishment of an animal model for decortication-facilitated orthodontics [5, 6, 12].

**Table 2:** The distances of tooth movement.

Tooth movement (n = 8)	Experimental groups (mm)	Control groups(mm)	P
1st	0.4662±0.072	0.4484±0.025	0.5196
3rd	0.5354±0.051	0.4866±0.023	0.0271*
5th	0.6404±0.038	0.5656±0.053	0.0059**
7th	0.6963±0.046	0.6418 ± 0.05	0.0396*
14th	0.8865±0.045	0.8016±0.045	0.0021**

*P < 0.05;

**P < 0.01

### The histological reaction in the periodontal tissue

**Fig 3:**
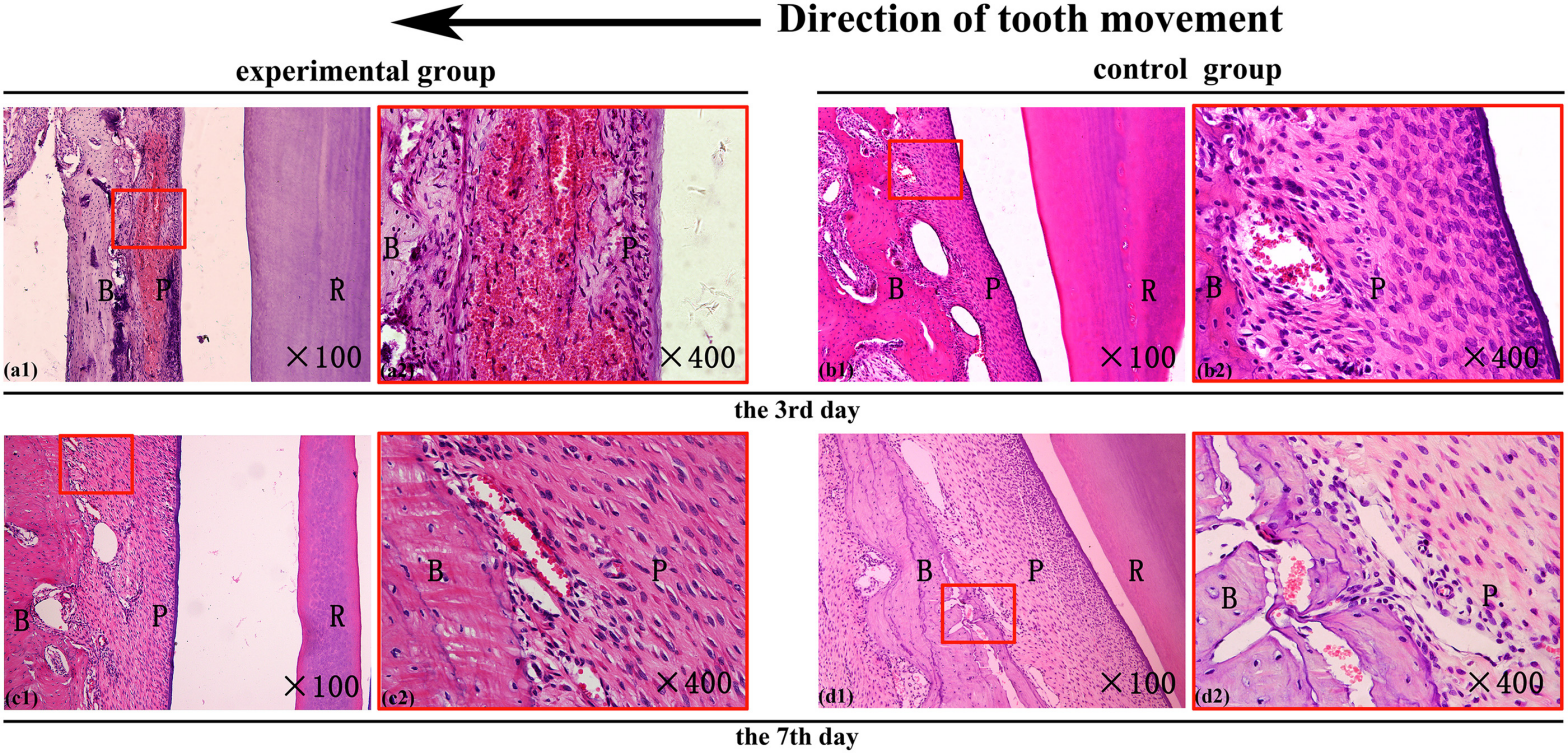
The histological reaction of the local periodontal tissues. H&E staining of the hematodes periodontal ligament and alveolar bone on the compressed side of the orthodontic tooth on the day 3 and day 7 after receiving orthodontics; R-tooth root, B-alveolar bone, P-periodontal ligament. The direction of the tooth movement is illustrated at the top (original magnification X100 on the left and X400 on the right).

The periodontal tissues were investigated with H&E staining in both groups on the days 3 and 7 after the procedure. Hyperemia of the periodontal ligament and the proximal alveolar wall was noted, particularly on day 3 (Fig 3). The hyperemia was more obvious in the experimental groups, who showed a large number of blood cells congested in the dilated capillary vessels, compared with the control groups. After day 7, however, that in the experimental groups was attenuated and the groups were similar in amount of hyperemia (Fig 3).

### Histological investigation of bone modeling

**Fig 4:**
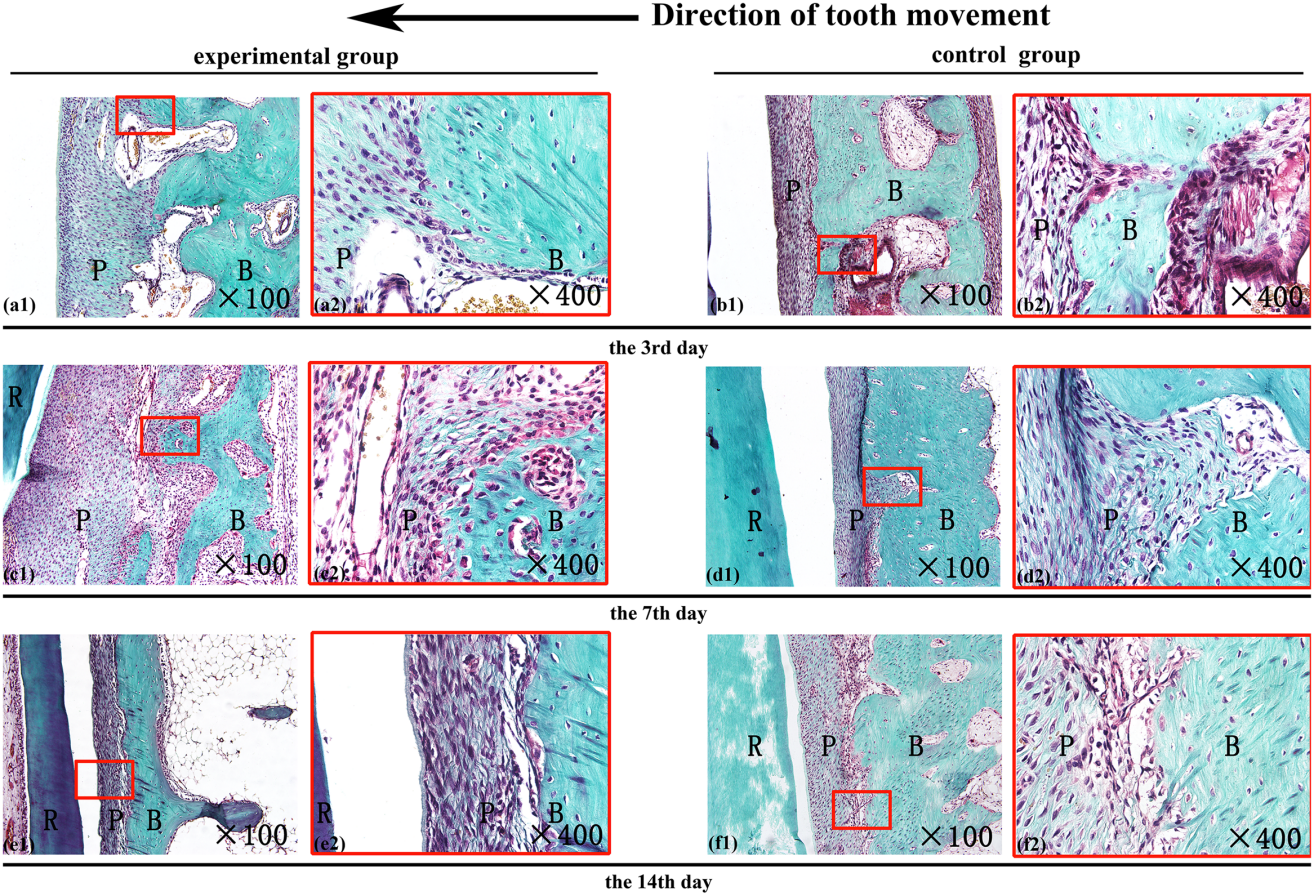
The effects of alveolar decortications on osteogenesis. Masson’s trichrome staining of the alveolar bone in the tensile side of the orthodontic tooth, on day 3, 7, and 14 days after receiving orthodontics. R-tooth root, B-alveolar bone, P-periodontal ligament. The direction of the tooth movement is illustrated at the top. (Original magnification X100 on the left, and X400 on the right).

New bone formation in the tensile sides were visualized with Masson's trichrome staining. In the periodontal tissues on the tensile sides, where osteogenesis tends to occur after 14 days [13], none of the groups (experimental or control) had obvious new osteoid formed on stained samples from days 3, 7 or 14. Furthermore, the connective tissue and the matured bone were similar between the experimental and control groups (Fig 4).

**Fig 5:**
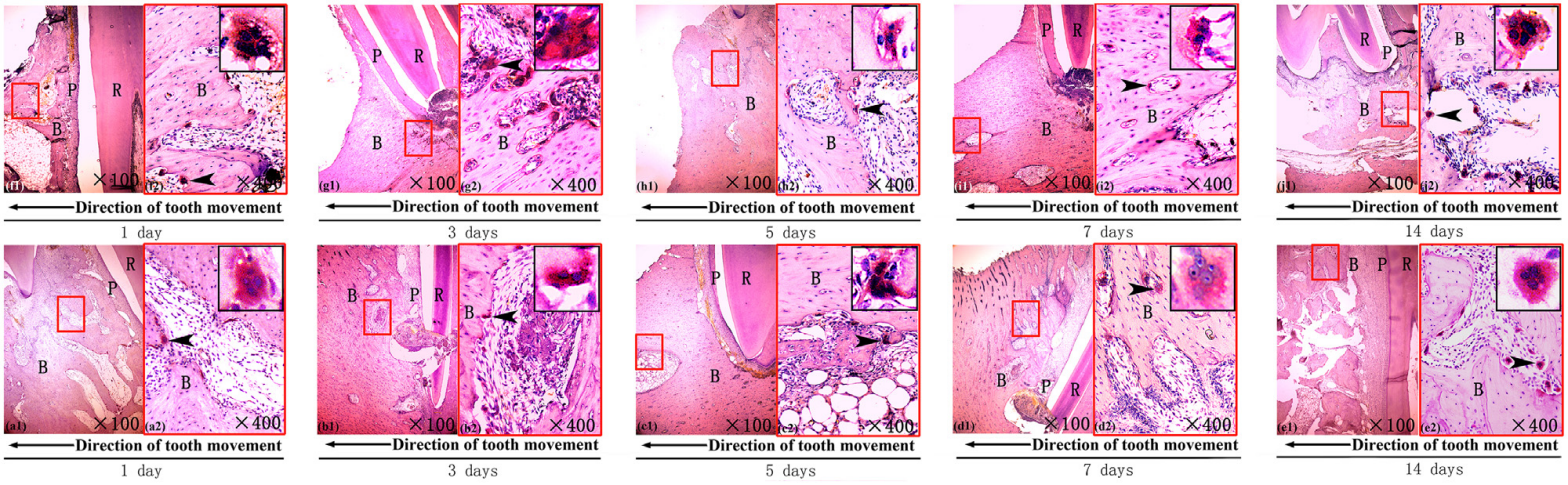
The effect of alveolar decortication on osteoclast formation. The periodontal tissues on the compressed sides of the teeth were stained with tartrate-resistant acid phosphatase (trap). **(a1-e2)** The osteoclasts in the experimental groups. **(f1-j2)** The osteoclasts in the control groups. R-tooth root, B-alveolar bone, P-periodontal ligament. The direction of the tooth movement is illustrated below each photograph. (Original magnification X100 on the left and X400 on the right). The representative osteoclast (arrowheads) is digitally magnified in the image at the top right corner of each photo.

The periodontal tissues in the compressed sides, where osteoclastogenesis is frequently observed [13], were investigated with trap staining. Trap-positive cells containing ≥3 nuclei were counted as osteoclasts [17, 18]. In contrast to the control groups, the decortication groups exhibited fairly pronounced osteoclastogenesis (Fig 5), especially by days 3, 7 and 14, when the osteoclast counts (trap + MNCs) were significantly greater for the experimental groups (P < 0.05; Fig 2; Table 3).

**Table 3:** The osteoclasts counting in the local alveolar bone.

Osteoclasts (n = 3)	Experimental groups	Control groups	P
1	34.33±10.75	22.46±5.61	0.1625
3	56.67±10.55	29.26±8.14	0.0235*
5	36.34±6.87	23.66±4.21	0.0527
7	41.56±7.25	25.34±5.48	0.0365*
14	52.33±8.58	36.67±6.54	0.0254*

*P < 0.05

Furthermore, the number of osteoclast count exhibited two peaks in the experimental groups: the first peak was on the day 3, after which the count fell close to its minimum value on the day 5. There was a subsequent rise from day 7 through day 14, when the count reached a second peak (Fig 2).

### The expression patterns of genes relating to bone remodeling

Isolation of mRNA from the compressed alveolar bone was performed and was analyzed for genes related to bone remodeling. In the experimental groups, the mRNA levels of trap and ctsk (cathepsin), markers reflecting osteoclast activity [19, 20], increased rapidly and reached maximum levels on the day 3, when they were significantly higher than those of the control groups (P < 0.05; Fig 2). Compared with day 1, the mRNA level in the experimental group on day 3 for trap and ctsk were greater by approximately 20-fold and 66-fold, respectively. In the control groups, however, they had increased by only four and fourteen-fold, respectively (Table 4).

**Table 4:** Relative expression of genes related to bone remodeling at different time points (day after surgery).

Trap (n = 5)	Experimental groups	Control groups	P
1st	0.4379±0.2782	0.5467±0.2417	0.5277
3rd	8.9119±4.8313	2.2194±1.2997	0.0173*
5th	0.9955±0.4552	2.4149±1.0865	0.0273*
7th	0.6765±0.3674	0.4674±0.3269	0.3696
14^th^	1.2051±0.6926	0.2639±0.1666	0.0188*
ctsk (n = 5)	experimental groups	control groups	P
1st	0.3548±0.2857	0.4591±0.2558	0.5599
3rd	23.2667±10.5222	6.5652±3.0743	0.0093**
5th	0.3834±0.2044	4.5391±2.0003	0.0017**
7th	0.6941±0.3389	0.2281±0.1109	0.0192*
14th	0.3329±0.1946	1.0701±0.6929	0.0512
nfatc1 (n = 5)	experimental groups	control groups	P
1st	1.8874±0.8866	1.0704±0.4943	0.1095
3rd	1.522±1.0405	0.6443±0.2967	0.1072
5th	0.7638±0.2757	0.5433±0.1667	0.1645
7th	4.1457±1.025	1.0087±0.5311	0.0003**
14th	1.9388±0.5686	0.6867±0.2451	0.0019**
fra2 (n = 5)	experimental groups	control groups	P
1st	1.1499±0.4042	1.4644±0.3037	0.2017
3rd	0.5259±0.2021	0.7529±0.1438	0.0749
5th	0.7394±0.3474	0.1012±0.085	0.004**
7th	3.4254±1.0142	2.2053±0.484	0.0413*
14th	5.0743±2.1238	1.624±1.0108	0.0112*
jdp2 (n = 5)	experimental groups	control groups	P
1st	0.8875±0.3895	0.5703±0.2943	0.1843
3rd	0.4222±0.1107	0.6114±0.1984	0.0996
5th	0.6638±0.4708	0.62±0.2377	0.8573
7th	3.8463±1.0225	2.2564±1.0311	0.0400*
14th	1.8381±0.8786	0.6986±0.3951	0.0295*
rankl/opg (n = 5)	experimental groups	control groups	P
1st	0.9133±0.3974	0.7866±0.5442	0.6852
3rd	0.1011±0.0424	0.1403±0.0656	0.2943
5th	4.985±1.1256	2.1699±1.295	0.0063**
7th	3.6463±1.3737	2.0897±0.4904	0.0441*
14th	4.6457±1.6246	2.3048±1.0925	0.0388*

* P < 0.05;

**P < 0.01

The mRNA levels of nfatc1 (nfat transcription complex 1) and jdp2 (jun dimerization protein 2), which reflect osteoclast differentiation [21, 22], were maintained at relatively low levels in the experimental groups until day 7, when they reached their maximum values and were significantly higher than the control groups (P<0.05; Fig 2). From day 5 to day 7, the mRNA of nfatc1 in the experimental group increased by approximately five-fold, and that of jdp2 by approximately six-fold; meanwhile, the control groups showed a less impressive rise of nfatc1 and jdp2 (less than 2 and 4-fold increases, respectively). Another marker of osteoclast differentiation, fra2 (fos-related antigen 2) [22], exhibited increased mRNA levels on the day 5 in the experimental group, and the levels continued to rise through day 14 (P<0.05; Fig 2; Table 4).

Similarly, in the experimental groups, the rankl/opg ratio, a positive indicator of osteoclastogenesis [23], was initially elevated on day 5. Subsequently, on days 5, 7 and 14, the rankl/opg ratios in the alveolar bone were significantly higher in the experimental groups than in the control groups (P<0.05; Fig 2; Table 4).

## Discussion

In this study, alveolar decortication-facilitated orthodontics and traditional orthodontics were performed in New Zealand white rabbits [24]. As in previous studies [12, 24], the mandibles of the rabbits were divided into decortication-facilitated (experimental) groups and traditional (control) groups (Fig 1). On days 3, 5, 7 and 14 after the orthodontic procedure, the tooth movement distances in the experimental groups were significantly greater than those in the control groups (Fig 2; Table 2), and this acceleration was consistent with decortication-facilitated orthodontic models described in previous studies [5, 6, 12].

As the decortications resulted in accelerated tooth movement, the compressed alveolar bone of the experimental groups developed significantly more osteoclasts relative to the controls (Figs 2 and 5). However, little new bone formation was observed in the tensile alveolar bones of both groups until the day 14, as illustrated by Masson's trichrome staining (Fig 4). According to previous studies, the differences of new bone formation rarely appeared between two groups in the first 14 days [25–27], which means the rate of osteogenesis has not accelerated by decortication. But, the bone resorption and osteoclast numbers were indeed up-regulated as early as 3 to 5 days post-procedure [28, 29]. Combined with previous studies [30], this suggests that the RAP (regional acceleratory phenomenon) may be induced by the decortications. According to our observation, alveolar decortication may induce osteoclasts more effectively than it does new bone formation early on after decortication. This circumstance may be the primary cause for the regional acceleratory phenomenon (RAP)—via decreasing the bone density and diminishing local resistance [5, 14].

Furthermore, the number of newly formed osteoclasts exhibited two peak counts—on the days 3 and 14 post-surgery (Figs 2 and 5). This was especially evident in the experimental groups, in which the peaks were highly significant. We observed a statistically greater number of osteoclasts in the experimental groups than in control groups on days 3, 7 and 14 (Fig 2). Furthermore, the two peak osteoclast counts coincided with the maximum mRNA level of different genes respectively, suggesting that the osteoclastogenic pattern was a result of different primary processes.

The mRNA levels of ctsk and TRAP reached their maximum values simultaneously with the first osteoclast peak on day 3 (Fig 2). Ctsk and TRAP, well-established biological markers of activation of newly formed osteoclasts [19, 20, 31] and which tend to reflect the maturation of precursors that may have existed previously, up-regulated on day 3. Meanwhile, the initial phase biomarkers of osteoclastogenesis (nfatc1 [nfat transcription complex1], jdp2 [jun dimerization protein 2] and fra2 [fos-related antigen 2]) still remained at relatively low levels at that time (Figs 2 and 5). Therefore, we speculate the first peak of osteoclastogenesis may have been the result of precursor activation, and the second peak due to differentiation of mononuclear cells which came from peripheral blood from the hyperemia that occurred after decortication.

Decortication led to hyperemia that persisted until day 7 (Fig 3), when the osteoclast count again increased significantly relative to the controls. Meanwhile, the mRNA levels of nfatc1 and jdp2 reached their maximum values, and the fra2 level began to increase continuously. On day 14, the osteoclast counts reached a second peak value (Figs 2 and 5). Although jdp2 can also be expressed in neutrophilic granulocytes or dendritic cells [32], we did not observe inflammation in the local tissues (Figs 3 and 5). During osteoclast differentiation, jdp2 combines with fra2 to form AP-1, which initiates osteoclasts differentiation [22, 33]. Meanwhile, nfatc1 regulates the transcription of ctsk and trap [21], which indicate osteoclast activity [19, 20]. Therefore, with the maximum levels of nfatc1, jdp2 and the up-regulation of fra2 on day 7, the subsequent rise in osteoclast counts may represent new differentiation of mononuclear cells from peripheral blood which came due to hyperemia [34].

The rankl/opg ratio, which is proportionate to the amount of osteoclastogenesis [23], was also up-regulated significantly from day 5 (Fig 2). This phenomenon also suggests new osteoclastogenic differentiation in the second peak count, rather than the maturation of precursors. The increased rankl/opg ratio may be attributable to the altered paracrine signaling of local stroma cells by decortications [17, 35, 36].

In this study, animal models of decortication-facilitated orthodontics were established for accelerated tooth movement, consistent with previous studies [5, 6, 12]. During the beginning stages, alveolar decortication appears to induce osteoclastogenesis significantly more than does new bone formation: this may be the primary process which leads to the regional acceleratory phenomenon (RAP). The results of this study also show decortication causes two phases of osteoclastogenesis-related mRNA elevation, which may have maintained the higher levels of osteoclast numbers which were found [8].
